# Molecular Biomarkers of Neovascular Age-Related Macular Degeneration With Incomplete Response to Anti-Vascular Endothelial Growth Factor Treatment

**DOI:** 10.3389/fphar.2020.594087

**Published:** 2020-12-29

**Authors:** Irmela Mantel, Angelica Borgo, Jacopo Guidotti, Edwige Forestier, Olga Kirsch, Yasmine Derradji, Patrice Waridel, Frédéric Burdet, Florence Mehl, Claude Schweizer, Raphaël Roduit

**Affiliations:** ^1^ Department of Ophthalmology, University of Lausanne, Jules-Gonin Eye Hospital, Lausanne, Switzerland; ^2^ Protein Analysis Facility, University of Lausanne, Lausanne, Switzerland; ^3^ Swiss Institute of Bioinformatics, Lausanne, Switzerland

**Keywords:** age-related macular degeneration (AMD), angiogenic factors, Inflammation, Soluble vascular cell adhesion molecule-1 (sVCAM-1), interleukin-6 (IL-6), bioactive interleukin-12 (IL-12p40), plasminogen ctivator inhibitor type 1 (PAI-1), hepatocyte growth factor (HGF)

## Abstract

The standard treatment for neovascular age-related macular degeneration (nAMD) consists of intravitreal anti-vascular endothelial growth factors (VEGF). However, for some patients, even maximal anti-VEGF treatment does not entirely suppress exudative activity. The goal of this study was to identify molecular biomarkers in nAMD with incomplete response to anti-VEGF treatment. Aqueous humor (AH) samples were collected from three groups of patients: 17 patients with nAMD responding incompletely to anti-VEGF (18 eyes), 17 patients affected by nAMD with normal treatment response (21 eyes), and 16 control patients without any retinopathy (16 eyes). Proteomic and multiplex analyses were performed on these samples. Proteomic analyses showed that nAMD patients with incomplete anti-VEGF response displayed an increased inflammatory response, complement activation, cytolysis, protein-lipid complex, and vasculature development pathways. Multiplex analyses revealed a significant increase of soluble vascular cell adhesion molecule-1 (sVCAM-1) [ *p* = 0.001], interleukin-6 (IL-6) [ *p* = 0.009], bioactive interleukin-12 (IL-12p40) [ *p* = 0.03], plasminogen activator inhibitor type 1 (PAI-1) [ *p* = 0.004], and hepatocyte growth factor (HGF) [ *p* = 0.004] levels in incomplete responders in comparison to normal responders. Interestingly, the same biomarkers showed a high intercorrelation with r2 values between 0.58 and 0.94. In addition, we confirmed by AlphaLISA the increase of sVCAM-1 [ *p* < 0.0001] and IL-6 [ *p* = 0.043] in the incomplete responder group. Incomplete responders in nAMD are associated with activated angiogenic and inflammatory pathways. The residual exudative activity of nAMD despite maximal anti-VEGF treatment may be related to both angiogenic and inflammatory responses requiring specific adjuvant therapy. Data are available via ProteomeXchange with identifier PXD02247

## Introduction

Age-related macular degeneration (AMD) is a multifactorial pathology involving a large number of molecular elements, such as growth factors, inflammatory molecules, proteins implicated in the complement system, fluid regulators, radicals and anti-oxidant factors, visual cycle products, and others (Lambert et al., 2016). Currently, treatment is available for the neovascular form of AMD (nAMD), consisting of intravitreal injections of anti-vascular endothelial growth factors anti-vascular endothelial growth factors (anti-VEGF). VEGF plays a key role in the pathogenesis of neovascularization and associated exudation (Spilsbury et al., 2000). Repeated injections of anti-VEGF are needed to suppress VEGF activity, prohibit further neovascular growth, and suppress exudation. Similar visual improvements have been achieved with ranibizumab (Brown et al., 2006; Rosenfeld et al., 2006), aflibercept (Heier et al., 2012), and bevacizumab (Martin et al., 2011).

The need for retreatment is variable, and injection frequency can be adapted to the individual’s requirements, which are guided mainly by signs of exudative activity. However, some patients with nAMD never achieve the normal response of complete fluid resolution, despite receiving anti-VEGF treatment every month (Fogli et al., 2018). These patients are often referred to as having refractory nAMD, showing pathologic intraretinal or subretinal fluid on optical coherence tomography (OCT) despite monthly treatment. The reasons for this incomplete response are unclear (Yang et al., 2016). However, because of the incomplete response to anti-VEGF, it is hypothesized that other molecular factors associated with exudative activity may be involved.

This study aimed to evaluate the molecular markers in the aqueous humor (AH) associated with nAMD, particularly in participants with incomplete response to anti-VEGF, and to correlate these markers with the treatment response to anti-VEGF treatment.

## Materials and Methods

This prospective study was performed at the Jules-Gonin Eye Hospital Medical Retina Unit and the basic science laboratory of the same institution, which are grouped within the Foundation Asile des Aveugles in Lausanne, Switzerland. The study protocol was approved by the local ethics committee (CER-VD, protocol ID 2017-02175). It adhered to all national legal requirements and the tenets of the Declaration of Helsinki. All participants provided written informed consent.

### Samples Selection

The study used AH from three groups of participants: patients affected by nAMD with incomplete response to anti-VEGF treatment (group R); patients affected by nAMD with complete fluid resolution on monthly or less frequent anti-VEGF treatment (group N); and control patients without any retinopathy (group C). The study protocol estimated that 20 participants in each group would allow for meaningful conclusions. A sample volume of 0.1 ml of AH was expected to be available for both proteomics and multiplex analysis.

To be eligible for group R, intraretinal and/or subretinal fluid had to be present on spectral-domain OCT (SD-OCT) at each monthly examination for at least 6 months, despite monthly intravitreal injections of anti-VEGF (ranibizumab or aflibercept). Anti-VEGF treatment had to be initiated and conducted without interruption for at least 12 months. An observe-and-plan regimen was used (Mantel et al., 2014). Following informed consent, between 30 and 100 µl of AH was collected, under topical anesthesia via paracentesis using a 26-gauge needle, immediately before a scheduled intravitreal injection of anti-VEGF. After collection, the AH samples were placed immediately in a −20°C freezer and, after 0–10 days, transferred to our institution’s biobank for storage at −80°C. Samples used in the study had been stored for up to 1.5 years.

Patients undergoing cataract surgery and giving informed consent for scientific use of biological waste materials were recruited prospectively and retrospectively (biobank) for groups N and C. AH was collected via the first paracentesis in the beginning of the cataract procedure. The following inclusion criteria applied: participants in Group N had nAMD on anti-VEGF treatment and complete dryness on SD-OCT (absence of intraretinal and subretinal fluid) achieved with monthly or less frequent retreatment or less frequently during the preceding 6 months. Group C had a normal retina and were only affected by cataracts. Patients with drusen AMD or any other retinopathy were excluded.

Exclusion criteria for all three groups included the following: any confounding retinopathy; diabetes (independent of presence or absence of diabetic retinopathy); insufficient visibility of the fundus for retinal diagnosis; any anterior segment eye surgery in the study eye within 3 months preceding AH collection; any posterior segment surgery within 6 months; any preceding treatment such as photodynamic treatment, laser, or intraocular or periocular steroids within the 6 months preceding AH collection; and inability to provide informed consent.

Complementary selection of AH samples from the biobank (group N and group C) was performed by the laboratory investigator (RR), guided by age-matched and sex-matched criteria for the prospectively recruited study participants in group R. No further clinical data regarding the coded data from the biobank were available to the laboratory investigator. However, the clinical investigator (IM) had full access to the patient files and imaging documentation. All files corresponding with the coded selected AH samples were validated by the clinical investigator according to inclusion and exclusion criteria.

In addition to the attributes of the study group, the following clinical data were included in the coded study database: age, sex, anti-VEGF treatment agent (ranibizumab or aflibercept) during 6 months preceding AH sample collection, and the date of the last injection before AH sample collection.

### Proteomic Analyses

#### Sample Preparation

Ten AH samples per group were processed in two separate batches of five for proteomic analyses. Proteins were digested according to a modified version of the iST protocol (Kulak et al., 2014). Fifteen microliters of modified iST buffer (2% sodium deoxycholate, 20 mM DTT, 5 mM EDTA, 200 mM Tris; pH 8.6) was added to 15 μl of AH sample and heated at 95°C for 5 min. Next, 10 μl of 160 mM chloroacetamide (in 10 mM Tris; pH 8.6) was added, and cysteines were alkylated for 45 min at 25°C in the dark. After 1:1 dilution with H_2_O, samples were digested with 0.2 μg of trypsin/Lys-C mix (Promega, Madison, WI, United states) and incubated at 37°C for 1 h, followed by the addition of a second enzyme (0.1 μg trypsin/LysC) and 1 h of incubation. To extract deoxycholate, two volumes of ethyl acetate plus 1% trifluoroacetic acid (TFA) were added to one volume of sample; then, the mixture was vortexed for 2 min and centrifuged for 2 min at 5,000 rpm*.* The bottom aqueous fraction was loaded onto an equilibrated OASIS MCX µElution plate (Waters, Milford, MA, United states) prefilled with SCX0 buffer (20% MeCN, 0.5% formic acid, 0 M ammonium acetate) and centrifuged*.* The columns were washed once with 200 μl ethylacetate plus 0.5% TFA and twice with 200 μl 2% MeCN plus 0.1% formic acid. Then, the peptide mixture was fractionated by sequential elution with 200 μl SCX200 buffer (20% MeCN, 0.5% formic acid, 200 mM ammonium acetate), 200 μl SCX500 buffer (20% MeCN, 0.5% formic acid, 500 mM ammonium acetate), and 200 μl basic elution buffer (80% MeCN, 19% water, 1% NH_3_).

#### Mass Spectrometry Analyses

Tryptic peptide fractions, prepared as described above, were dried, and resuspended in 0.05% trifluoroacetic acid and 2% (v/v) acetonitrile for mass spectrometry (MS) analyses. Tryptic peptide solutions were injected using an Ultimate RSLC 3000 nano high-performance liquid chromatography (HPLC) system (Dionex, Sunnyvale, CA, United states) interfaced with an Orbitrap Fusion Tribrid mass spectrometer (Thermo Scientific, Bremen, Germany). Peptides were loaded onto a trapping microcolumn Acclaim PepMap100 C18 (20 mm × 100 μm inner diameter [ID], 5 μm particles, 100 Å; Thermo Scientific) before separation on a reverse-phase custom packed nanocolumn (75 μm ID × 40 cm, 1.8 μm particles; Reprosil Pur; Dr Maisch GmbH, Ammerbuch, Germany). A flow rate of 0.25 μl/min was used with a gradient of 4%–76% acetonitrile in 0.1% formic acid (total time: 65 min). Full survey scans were performed at a resolution of 120,000, and a top speed precursor selection strategy (Hebert et al., 2013) was applied to maximize the acquisition of peptide tandem mass spectrometry (MS/MS) spectra with a maximum cycle time of 0.6 s. The HCD fragmentation mode at a normalized collision energy of 32% and a precursor isolation window of 1.6 m/z were used; MS/MS spectra were acquired in the ion trap. Peptides selected for MS/MS were excluded from further fragmentation for 60 s.

#### Data Analysis

Tandem MS data were processed using MaxQuant software (version 1.6.3.4) (Cox and Mann, 2008), incorporating the Andromeda search engine (Cox et al., 2011). The UniProt human reference proteome database for January 2019 was used (73,950 sequences) and supplemented with sequences of common contaminants. Trypsin (cleavage at K and R) was used as the enzyme parameter, allowing two missed cleavages. Carbamidomethylation of cysteine was specified as a fixed modification. N-terminal acetylation of protein and oxidation of methionine were specified as variable modifications. All identifications were filtered at a 1% false discovery rate (FDR) at both the peptide and protein levels with default MaxQuant parameters. For protein quantitation, either the iBAQ (Schwanhäusser et al., 2011) or the LFQ label-free values (Cox et al., 2014) were used. MaxQuant data were further processed with Perseus software (Tyanova et al., 2016) for filtering, log2 transformation, normalization of values, statistical analyses, and Gene Ontology (GO) annotations.

### Multiplex Analysis

A multiplex analysis was performed using Luminex xMAP technology (ProcartaPlex; Thermo Fisher Scientific, Waltham, MA, United states), which is a bead-based immunoassay in microplate format. This system allows simultaneous detection of many cytokines, chemokines, growth factors, and other protein targets from humans in very small fluid volumes. Each target molecule has its own standard curve, allowing a specific quantification whose sensitivity and limit of detection changes according to the metabolite. The target proteins for our study were identified according to previous publications suggesting a role within AMD (de Oliveira Dias et al., 2011; Jonas et al., 2012; Nassar et al., 2013; Knickelbein et al., 2015; Liu et al., 2016; Sato et al., 2018). This composed our test plate ProcartaPlex 1. We first tested three volumes of AH (5, 10, and 20 μl) from two control patients to assess the detection limit and the linearity of response for each analyte (Supplementary Figure S1A). In addition, it allowed for defining the test volume needed for each analyte. In the next step, we tested ten AH samples per group for those analytes with a confirmed linear response (Supplementary Figure S1B: ProcartaPlex 1). A second round of analysis was performed with a newly composed test plate (Supplementary Figure S1C: ProcartaPlex 2), adding some new analytes to the confirmed linear response analytes and using those samples with sufficient remaining fluid. To detect all 23 analytes according to the manufacturer’s protocol, 10 µl of AH (in duplicate) were used.

### AlphaLISA Assays

Homogenous no-wash immunoassays (AlphaLISA; PerkinElmer, Waltham, MA, United states) were used to confirm the most promising results of the multiplex analysis (human VEGF-A: AL201; human VCAM-1: AL338; human IL-6: AL3025). We aimed to perform the AlphaLISA for up to 10 samples, but this was dependent on the residual AH volume available after performing the previous steps. We used 2–5 μl (in triplicate) of AH to perform the assay. The AlphaLISA assay contained acceptor beads coated with an anti-analyte antibody, streptavidin-coated donor beads, a biotinylated anti-analyte antibody, a lyophilizate analyte (for the standard curve), and an assay buffer (10×). After incubation with all components, quantification of the signal was performed using an EnVision multimode plate reader (PerkinElmer).

### Clinical Phenotypes

The clinical phenotype of nAMD in eyes included into groups N and R were determined based on SD-OCT, infrared images, and dye angiography (fluorescein and indocyanine green). An experienced retinal specialist (IM) determined the type of neovascular membrane, the presence of reticular pseudodrusen, the presence of complete/incomplete retinal pigment epithelium and outer retinal atrophy (iRORA and cRORA), the presence of major fibrotic changes, central retinal thickness, and subfoveal choroidal thickness, and the type of exudative fluid of the recurrences (presence of intraretinal fluid, presence of subretinal fluid).

### Bio-Informatic Analyses

Statistics were performed using protein quantitation iBAQ values in the R statistical programming environment (version 3.6.1; https://www.r-project.org/). For analysis of the proteomics results, linear models (R function lm) adjusted for batches were used to identify differentially expressed proteins (*p* < 0.1) to compare group N and group R and to compare groups C/N and group R. For these, overrepresentation tests were performed using the Bioconductor (http://www.Bioconductor.org) package ClusterProfiler (Yu et al., 2012). The functions “enrichGO” and [OrgDb = org. Hs.e.g., db, keyType = “ENTREZID,” ont = “ALL,” pAdjustMethod = “BH,” pvalueCutoff = 0.1, qvalueCutoff = 0.1, readable = T] were used as the parameters to test whether genes mapped on selected proteins were significantly overrepresented in the given GO terms.

Gene Set Enrichment Analysis (GSEA) software (version 4.0.2; http://software.broadinstitute.org/gsea) was used along with the MSigDB annotation c5 (version 7.0; GO ontology); both of which were provided by the Broad Institute. The minimum size of the GO term was set to 15, and the maximum size was set to 500. One thousand permutations were performed to assess the empirical FDR. The first result of the GSEA was the enrichment score (ES), which reflects the degree to which a gene set is overrepresented at the top or bottom of a ranked list of genes. GSEA calculates the ES by walking down the ranked list of genes and increasing a running-sum statistic when a gene is in the gene set and decreasing it when it is not. The normalized enrichment score accounts for differences in the gene set size and in correlations between gene sets and the expression dataset; it is the primary statistic for examining gene set enrichment results.

For analysis of the multiplex results, the continuous concentration results were compared between groups using an ANOVA test with Holm–Sidak’s correction for the three comparisons per analyte. For correction of multiple molecules being tested with multiplex analysis, a false discovery rate control was applied (Benjamini and Hochberg, 1995).

The relevant molecular biomarkers were analyzed for their association with the phenotypic features: A correlation analysis was performed for continuous parameters, and an ANOVA test for categorical parameters.

## Results

### Clinical Characteristics

The clinical characteristics of the groups are summarized in Table 1. The nAMD groups R and N were similar in terms of age and gender distribution. The neovascular phenotype distribution was balanced, including a high proportion of type 1 neovascularization in both groups. The reticular pseudodrusen and presence of fibrosis were equally distributed, and the mean subfoveal choroidal thickness was similar. However, cRORA and iRORA, as well as intraretinal fluid being implicated in the exudative manifestations were more frequent in group N, whilst subretinal fluid and thicker central retinal thickness was more frequently found in group R.

**TABLE 1:** Demographic, phenotypic and treatment characteristics of each group based on the type of molecular analysis.

	rAMD (R)	nAMD (N)	Control (C)
*N* patients	17	17	16
Mean age (SD)	81.6 (±6.5)	81.6 (±5.9)	70.44 ± 2.47
Females (%)	13 (76.5%)	13 (76.5%)	8/8
*N* eyes	18	19	16
Right eyes/left eyes	14/4	7/13	10/6
Neovascularization type: 1/2/3	15/2/1	16/3/1	—
Presence of reticular pseudodrusen (%)	5 (28%)	7 (35%)	—
Presence of fibrosis (%)	3 (17%)	4 (20%)	—
Presence of cRORA	4 (22%)	10 (50%)	—
Presence of iRORA	6 (33%)	14 (70%)	—
Recurrences including IRF (%)	9 (50%)	17 (85%)	—
Recurrences including SRF (%)	12 (67%)	8 (40%)	—
Mean CRT in micrometers (SD)	323 (±83)	286 (±76)	—
Mean subfoveal choroidal thickness in micrometers (SD)	150 (±121)	166 (±64)	—
Treatment duration in months ± SD	69.7 ± 35.1	55.4 ± 36.2	—
Number of injections received ± SD	59.6 ± 29.9	36.4 ± 26.7	—
Weeks from preceding injection ± SEM	5.89 ± 0.94	3.97 ± 0.29	—
Anti-VEGF agent used (ranib./Aflib.)	8/10	8/12	—
Number of switches performed			
0	3	13	—
1	9	7	—
2	6	0	—
Mean visual acuity at treatment initiation in ETDRS letters ± SD	61.7 ± 12.5	63.1 ± 16.1	—
Mean visual acuity change from baseline in ETDRS letters ± SD	7.9 ± 12.5	Before cataract surgery −9.4 ± 12.9 after cataract surgery 12.8 ± 11.8	—

cRORA, complete retinal pigment epithelium pigment and outer retinal atrophy; iRORA, incomplete retinal pigment epithelium pigment and outer retinal atrophy.

The mean treatment duration was long in both groups, but even more so for group R. In parallel, the number of injections received preciously was more elevated in group R. The medication used during the months before inclusion into the study was similarly distributed between aflibercept and ranibizumab in the groups R and N. Treatment switches had been performed between anti-VEGF agents more frequently in group R. The visual benefit from treatment initiation was good in both groups, more so for group N after cataract surgery.

### Proteomics Analysis

#### Anti-Vascular Endothelial Growth Factors-A Quantitation

**FIGURE 1:**
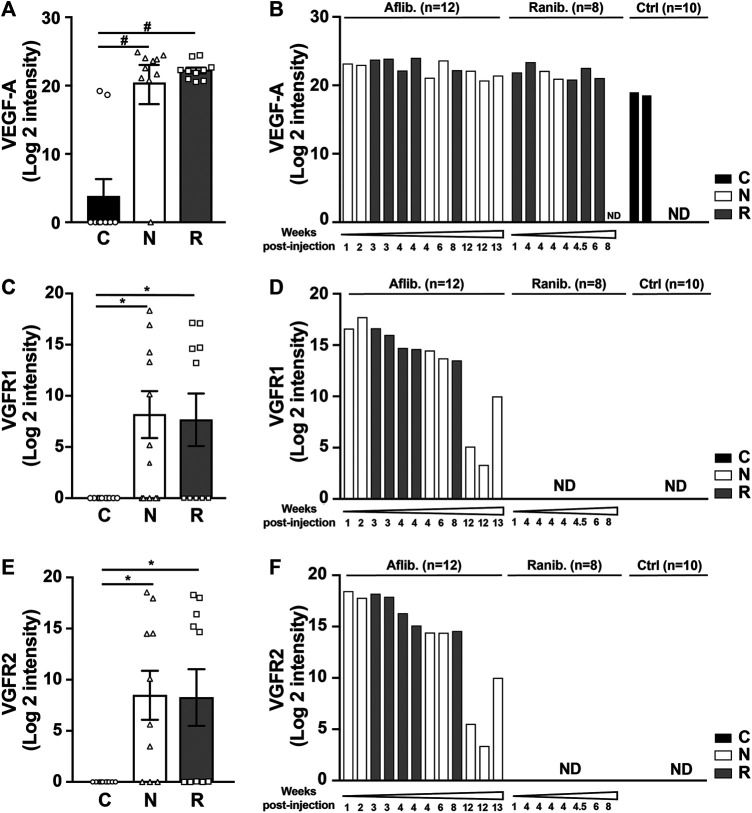
Proteomic analyses of ten AH from each group of patient nAMD (N), rAMD (R), and controls (C). Graphic representation of VEGF-A **(A)**, VEGFR1 **(C)** and VEGFR2 **(E)** levels obtained in the three different study groups. *p < 0.03 and #*p* < 0.0001 (Left). Graphs on the right represent the quantification of VEGF-A **(B)**, VEGFR1 **(D)** and VEGFR2 **(F)** independently of the type of AMD but depending on the anti-VEGF treatment (aflibercept [Aflib] or ranibizumab [Ranib]) and classify according to the time after the last injection of anti-VEGF inhibitors.

The VEGF-A concentrations were found to be increased in both nAMD groups (groups R and N) (Figure 1A, left). Within the nAMD groups, this increase was independent of the drug type used and the time since the last injection (Figure 1A, right). The expected general increase in VEGF-A served as validation of the proteomics approach for AH. Additionally, the N-terminal parts of the VEGF receptor-1 (VEGFR-1/FLT-1) and VEGF receptor-2 (VEGFR-2/FLK-1) were also found in higher concentrations in both nAMD groups (Figure 1B, left). These components are part of the aflibercept molecules, and we consider this result to be a direct effect of aflibercept injections, as they were discovered only in Aflibercept-treated patients (Figure 1B, right). No other sequences of VEGFR-1 and VEGFR-2 proteins, except the ones present in aflibercept, were detected by the proteomics analyses. All patients treated with this drug showed increased VEGFR-1 and VEGFR-2 up to 2 months after the last injection, with a decrease after 3 months of aflibercept treatment; however, no detection of this compound was observed in ranibizumab-treated patients or in the control group (Figure 1B, right).

#### Gene Ontology Enrichment

**FIGURE 2:**
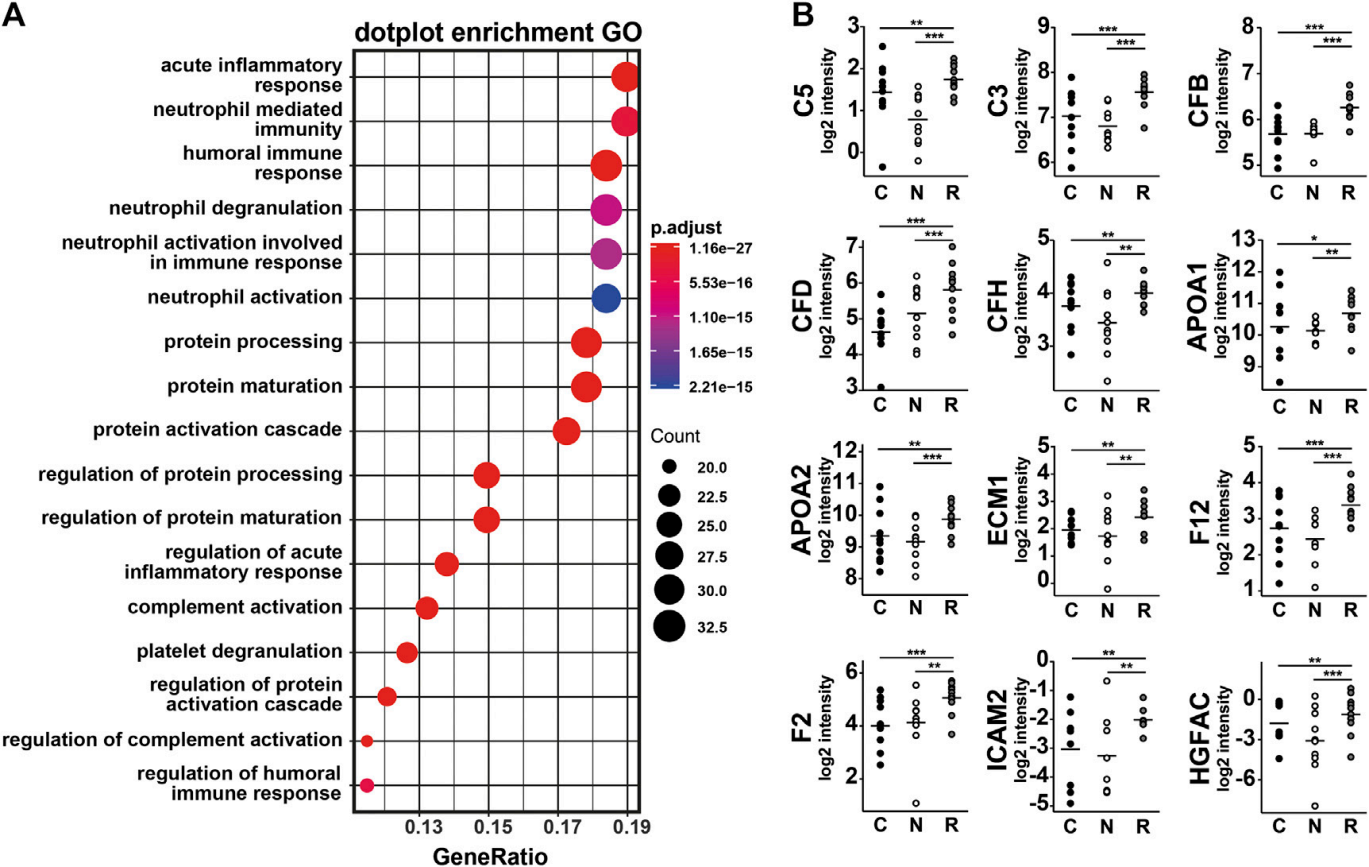
Proteomic bio-informatic analyses of ten samples per study group. **(A)** Dot plot showing GO enrichment. Statistical significance of the enriched terms is based on the *p*-values corrected by multiple testing (color-coded). The number of genes associated with each respective term is indicated by the dot size. **(B)** Boxplots expression of genes involved in GO are highlighted in red in **(A)** with significance levels (*p < 0.1, **p < 0.05, and ***p < 0.01) obtained from linear models comparing N and R and comparing C/N and R.

**FIGURE 3:**
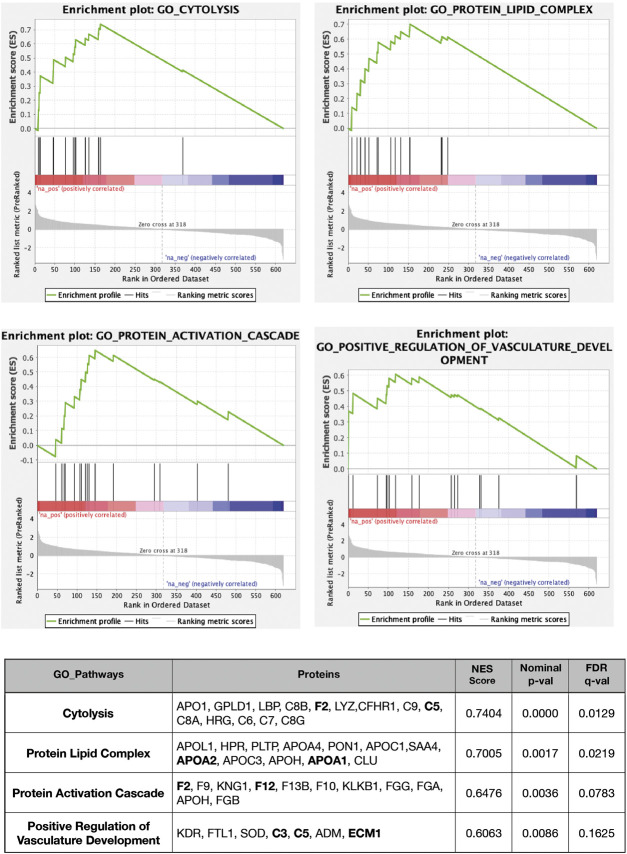
GSEA-based GO analysis enrichment plots of representative gene sets for several pathways. The primary result of the gene set enrichment analysis is the enrichment score (ES, at the top of the green curve), which reflects the degree to which a gene set is overrepresented at the top or bottom of a ranked list of genes. GSEA calculates the ES by walking down the ranked list of genes, increasing a running-sum statistic when a gene is in the gene set and decreasing it when it is not. The magnitude of the increment depends on the correlation of the gene with the phenotype. The ES is the maximum deviation from zero encountered in walking the list. A positive ES indicates gene set enrichment at the top of the ranked list; a negative ES indicates gene set enrichment at the bottom of the ranked list. The middle portion of the plot shows where the members of the gene set appear in the ranked list of genes. The bottom part of the plot shows the values of the estimates of the linear model. Genes included in GO pathways, normalized enrichment scores, nominal *p*-values (from 1,000 permutations), and FDR q-values are indicated in the table. The complete lists of the GSEA output are provided for positive and negative enrichment. For more details, see the supplemental Excel table.

Graphical heatmap representation of proteomics results using ten AH per group (Supplementary Figure S2). The GSEA of proteomics data comparing group R to groups N and C detected an increase in several pathways, including the acute inflammatory response, protein activation cascade, complement activation, and proteins involved in coagulation (Figure 2A). The concentration measurements for each group showed no differences in expression or trends of low expression of specific proteins in group N when compared to group C. However, some proteins were clearly increased in group R (Figure 2B), and this increase was statistically significant for complement C5, complement C3, complement factor B (CFB), complement factor D (CFD), complement factor H (CFH), apolipoprotein A1 (APOA1), apolipoprotein 2 (APOA2), endoplasmic reticulum membrane protein complex (EMC1), coagulation factor 12 (F12), coagulation factor 2 (F2), intercellular adhesion molecule 2 (ICAM2), and hepatocyte growth factor activator (HGFAC). A GSEA based on the rank lists from the linear model analysis showed that several additional proteins involved in cytolysis, the protein activation cascade, the lipid complex, and regulation of vasculature development were increased in group R (Figure 3). Whole file with GO analyses are provided in Supplementary Data Sheet S1.

### Multiplex Analysis

Within the multiplex analysis, the linearity response of the selection of 30 analytes was evaluated within the control group. The study revealed 15 analytes with a linear and reproducible response, six analytes that were partially detected with no linearity, and nine analytes that were not detected in 10 μl of AH (Supplementary Figure S1A). We modified our panel by removing undetected analytes and adding new ones (Supplementary Figure S1B); this allowed for the detection of 8 additional analytes with a reliable response. Table 2 shows the results obtained for 23 reliable analytes for all three groups of patients, the number of AH samples tested for each group, and the *p*-values obtained according to the analysis of variance (ANOVA). The numbers of test results per analyte varied, due to total available AH volume was often smaller than the expected 0.1 ml, some volume was used for proteomics, and some tests failed. The numbers of successfully performed test per analyte are given in Table 2 (between brackets after each analyte).

**TABLE 2:** Multiplex analysis of AH from nAMD patients with normal response (N), incomplete response (R) patients, and controls (C).

	Control C (*n*)	nAMD N (*n*)	rAMD R (*n*)	*p* values		
				*C* vs. *N*	*C* vs. *R*	*R* vs. *N*
VEGFR-1	100 ± 15.58 (9)	4,569 ± 1,228 (13)	4,738 ± 1,216 (12)	*0.024	*0.024	0.910
VEGFR-2	100 ± 5.85 (9)	172.1 ± 18.46 (13)	220.8 ± 28.66 (12)	0.055	*0.002	0.110
VEGF-A	100 ± 9.53 (14)	714 ± 142.8 (19)	712.4 ± 125.6 (18)	**0.003	**0.003	0.992
EGF	100 ± 2.14 (10)	105 ± 3.26 (10)	110.5 ± 1.71 (10)	0.242	*0.016	0.242
Eotaxin	100 ± 12.57 (13)	165 ± 23.5 (19)	238.8 ± 41.1 (18)	0.154	*0.011	0.151
HGF	100 ± 10.03 (14)	129.4 ± 10.82 (19)	232 ± 35.43 (18)	0.388	**0.001	**0.004
IL-12p40	100 ± 7.40 (14)	155.9 ± 14.15 (19)	210 ± 20.47 (18)	*0.031	**#0.0001**	*0.031
sVCAM-1	100 ± 8.93 (14)	122.8 ± 12.63 (19)	242.7 ± 35.69 (18)	0.511	**#0.0005**	**0.001
IL-6	100 ± 25.70 (6)	86.12 ± 64.25 (7)	10,546 ± 7,320 (10)	*0.042	0.11	**0.009
IP-10	100 ± 16.91 (14)	235.5 ± 42.06 (19)	280.2 ± 62.2 (18)	0.107	*0.038	0.490
MCP-1	100 ± 6.46 (14)	137.8 ± 11.16 (19)	154.3 ± 11.35 (18)	*0.032	**0.002	0.253
MMP-9	100 ± 18.31 (13)	233.3 ± 42.79 (19)	325.4 ± 64.23 (18)	0.144	*0.011	0.174
Adiponectin	100 ± 33.65 (4)	131.7 ± 32.36 (9)	173.2 ± 60.66 (8)	0.762	0.741	0.762
Eotaxin-3	100 ± 18.98 (5)	145.9 ± 27.1 (9)	120.3 ± 19.7 (10)	0.781	0.857	0.781
Ghrelin	100 ± 5.83 (4)	105.3 ± 5.74 (9)	103 ± 8.74 (8)	0.960	0.960	0.960
IL-3	100 ± 25.06 (5)	122.7 ± 36.17 (4)	110.5 ± 23.78 (5)	0.946	0.946	0.928
IL-7	100 ± 18.25 (8)	290.5 ± 44.59 (12)	360.1 ± 40.22 (11)	**0.006	**#0.0005**	0.203
MIF	100 ± 17.19 (4)	138.9 ± 20.84 (9)	133.5 ± 20.95 (8)	0.619	0.619	0.848
NGAL	100 ± 19.1 (9)	120.1 ± 10.53 (13)	135.4 ± 13.54 (12)	0.451	0.187	0.451
PaI-1	100 ± 8.5 (12)	179.9 ± 18.39 (13)	425.7 ± 89.64 (13)	0.304	**#0.0005**	**0.005
Gro-alpha-KC	100 ± 8.27 (14)	94.71 ± 5.60 (19)	162.7 ± 42.79 (17)	0.887	0.199	0.1669
IL-1RA	100 ± 32.02 (12)	70.77 ± 14.78 (16)	149 ± 61.91 (14)	0.652	0.652	0.4121
IL-13	100 ± 0.90 (10)	96.14 ± 3.46 (10)	97.66 ± 2.16 (10)	0.608	0.749	0.749

The number of samples tested for each group of patient is mentioned between brackets after each analyte (n). Results are expressed as mean ± SEM and as % of the control (^*^p < 0.05, ^*^^*^p < 0.005, and ^#^p < 0.0005, using ANOVA Holm-Sidak’s multiple comparisons test per analyte). Significant values are showed in bold.

First, the multiplex results were analyzed to confirm the results obtained by the proteomic analyses. A significant increase in VEGF-A, VEGFR-1, and VEGFR-2 was observed in both group R and group N (Table 2) without a significant difference between these two groups. The VEGFR components were detected in aflibercept-treated patients only (Supplementary Figure S3A).

**FIGURE 4:**
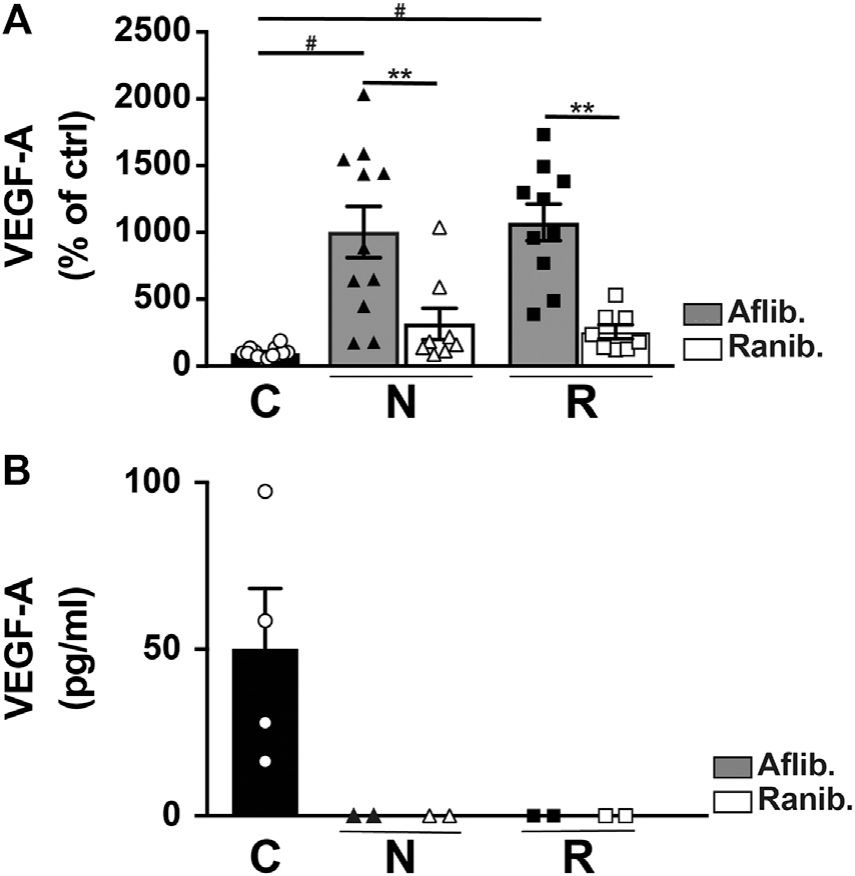
Level of VEGF-A in AH from nAMD patients with normal response (N), incomplete response (R), and controls (C). The VEGF-A was assessed by multiplex **(A)** or AlphaLISA **(B)** assays, and the results expressed are dependent on the anti-VEGF treatment (aflibercept [Aflib] or ranibizumab [Ranib]). **p < 0.0003 and #*p* < 0.0001.

VEGF-A levels were lower in patients treated with ranibizumab than with aflibercept when using multiplex analysis (Figure 4A), but they were both undetectable in AlphaLISA (Figure 4B). In contrast, VEGF-A was detected in control participants (Figure 4B). Additional testing of control AH after the addition of ranibizumab or aflibercept showed apparent interference with the detection of VEGF-A, thereby reducing its measurement significantly (Supplementary Figures S3B, C).

**FIGURE 5:**
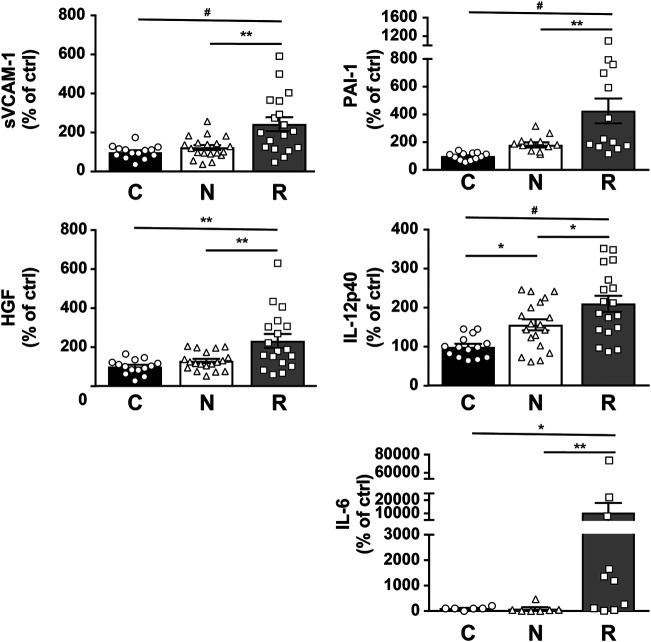
Multiplex analysis of AH from nAMD patients with normal response (N), incomplete response (R), and controls (C). Graphic representation of sVCAM-1, PAI-1, HFG, IL-12p40, and IL-6. Results are expressed as mean ± SEM and as % of the control group (*p < 0.05, **p < 0.005, and #*p* < 0.0005).

**FIGURE 6:**
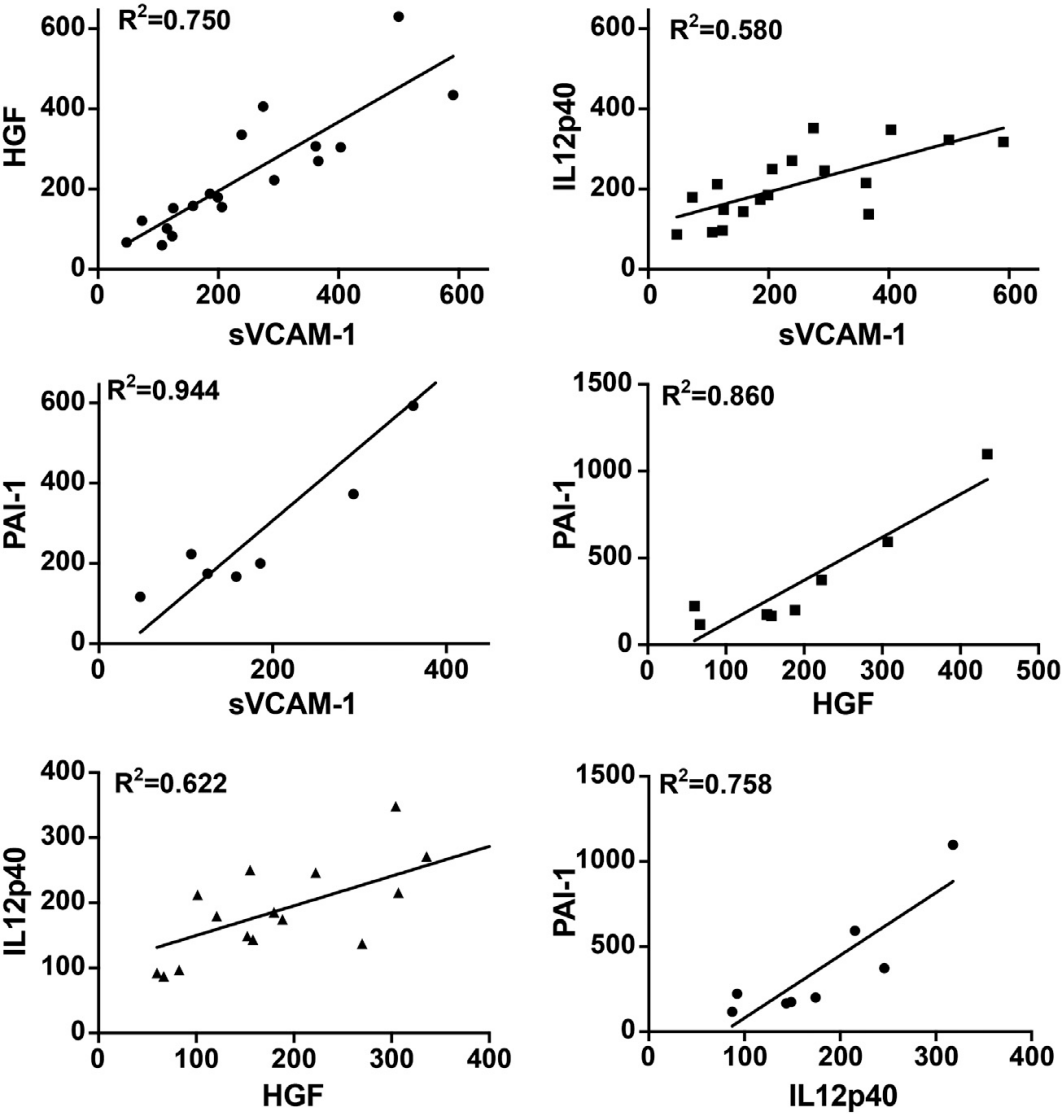
Correlation between different AH biomarkers in group R with incomplete anti-VEGF response. Levels of 4 biomarkers were compared in AH of incomplete responders. A linear regression (*R* ^2^) was measured in all comparisons. sVCAM-1 vs. PAI-1: *R* ^2^ = 0.944; sVCAM-1 vs. HGF: *R* ^2^ = 0.750; sVCAM-1 vs. IL-12p40: *R* ^2^ = 0.508; HGF vs. PAI-1: *R* ^2^ = 0.806; HGF vs. IL-12p40: *R* ^2^ = 0.622; IL-12p40 vs. PAI-1: *R* ^2^ = 0.758.

Several molecules, including soluble vascular cell adhesion molecule-1 (sVCAM-1), hepatocyte growth factor (HGF), plasminogen activator inhibitor type 1 (PAI-1), interleukin-12 subunit beta (IL-12p40), and interleukin-6 (IL-6), were significantly increased in group R compared to group C and group N (Table 2; Figure 5). The increase of these molecules was observed in both ranibizumab- and aflibercept-treated eyes, and the difference was non-significant between differentially treated patients (Supplementary Figure S4A). To confirm this, we performed an AlphaLISA for sVCAM-1 and observed a significant increase in both ranibizumab- and aflibercept-treated patients of group R in comparison to groups C and N (*p* < 0.0001). We also compared multiplex and AlphaLISA assays for the detection of sVCAM-1 and observed a correlation coefficient (*r* ^2^ = 0.924) suggesting that both assays are suitable and comparable (Supplementary Figure S5A). We also tested IL-6 levels using AlphaLISA, and we confirmed the increasing level of cytokines in group R in comparison to groups C and N (*p* = 0.043) (Supplementary Figure S5B). Figure 6 shows the correlation between four of these biomarkers (sVCAM-1 vs. PAI-1: *R* ^2^ = 0.944; sVCAM-1 vs. HGF: *R* ^2^ = 0.750; sVCAM-1 vs. IL-12p40: *R* ^2^ = 0.580; HGF vs. PAI-1: *R* ^2^ = 0.860; HGF vs. IL-12p40: *R* ^2^ = 0.622; IL-12p40 vs. PAI-1: *R* ^2^ = 0.758) (Figure 6), while no correlation was observed between these four biomarkers and IL-6 (data not shown).

Multiple testings of the false discovery rate control confirmed the statistical significance for four out of the five discussed proteins: the reference *p* values for the 23 molecules included in multiplex testing are 0.002, 0.004, 0.007, 0.009, and 0.011 for the first, second, third, fourth and fifth significant result, respectively. Thus, sVCAM (*p* = 0.001), PAI-1 (*p* = 0.004), HGF (*p* = 0.004), and IL-6 (*p* = 0.009) do fulfill the false discovery rate control but not IL-12p40 does not (*p* = 0.031).

**FIGURE 7:**
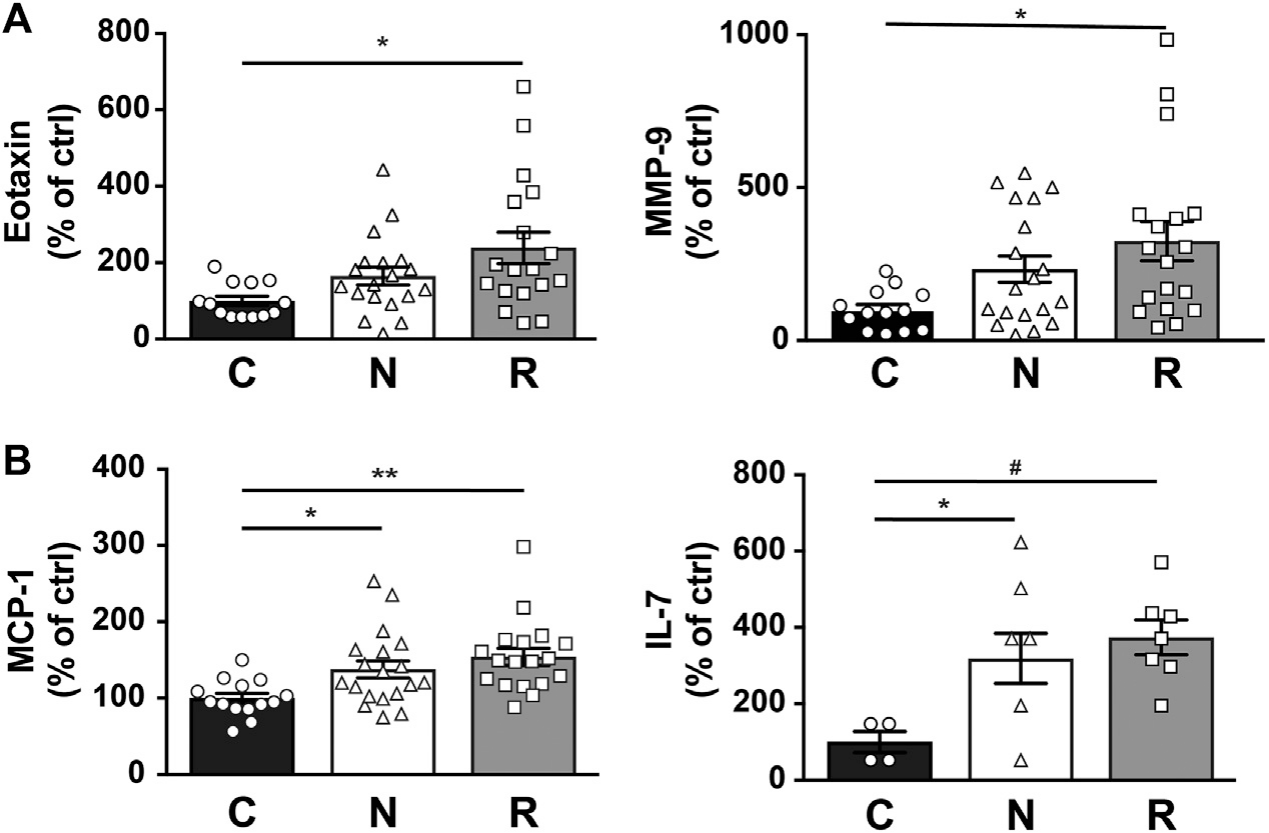
Multiplex analysis of AH from nAMD patients with normal response (*N*), incomplete response (R), and controls (C). Graphic representation of EOTAXIN and MMP-9 **(A)** and of MCP-1 and IL-7 **(B)** levels in the study groups. Results are expressed as mean ± SEM and as % of the control (*p < 0.05, **p < 0.005, and #*p* < 0.0005).

The Eotaxin chemokine and the matrix metalloproteinase 9 (MMP-9) showed increasing values from group C to group N to group R. However, the difference was statistically significant only for the comparison between group R and group C (Figure 7A). The results were not affected by the 2 different treatments (Supplementary Figure S4B). Both monocyte chemoattractant protein 1 (MCP-1) and interleukin 7 (IL-7) were significantly increased in both group R and group N (Figure 7B). For MCP-1, the increase was independent of the anti-VEGF drug used. However, IL-7 appeared to increase, specifically in aflibercept-treated patients (Supplementary Figure S4C).

### Exploratory Clinical-Molecular Correlation

The relevant molecular biomarkers that were associated with incomplete anti-VEGF response (group R: sVCAM, PAI-1, HGF, and IL-6, IL-12p40) were finally analyzed for potential associations with clinical characteristics. The results are summarized in Table 3.

**TABLE 3:** Clinical-Molecular Association Analysis: The five relevant molecules, associated with incomplete anti-VEGF response, were explored for potential associations with clinical parameters, in all neovascular age-related macular degeneration (nAMD) eyes included. Correlation analysis was performed for continuous variable, and Anova test for categorical variables.

	sVCAM-1		IL-12p40		IL-6		PAI-1		HGF		VEGF-A	
Continuous variables	r2	*p*	r2	*p*	r2	*p*	r2	*p*	r2	*p*	r2	*p*
Age	0.20	0.22	0.32	**0.05**	0.17	0.29	0.24	0.13	0.38	**0.01**	0.07	0.67
*N* anti-VEGF injections	0.26	0.10	0.30	0.06	0.26	0.11	0.13	0.42	0.26	0.10	0.13	0.42
*N* months of treatment	0.14	0.40	0.21	0.19	0.25	0.10	0.08	0.60	0.15	0.35	0.09	0.60
VA change from baseline	0.18	0.25	0.01	0.93	−0.35	**0.03**	0.16	0.32	0.18	0.27	−0.11	0.47
Central retinal thickness	0.19	0.24	0.29	0.07	0.04	0.80	0.26	0.12	0.18	0.26	0.04	0.82
Subfoveal choroidal thickness	−0.40	**0.01**	−0.46	**0.003**	−0.27	0.10	−0.26	0.10	−0.44	**0.005**	−0.14	0.38
Categorical variables	p		p		p		p		p		p	
Reticular pseudodrusen	0.38		0.69		0.36		0.56		0.86		0.58	
Intraretinal fluid	0.74		0.87		0.22		0.41		0.8		0.55	
Subretinal fluid	0.73		0.4		0.42		0.99		0.93		0.69	
cRORA	0.83		0.7		0.36		0.36		0.7		0.51	
iRORA	0.97		0.61		0.42		0.89		0.71		0.65	
CNV type	0.1		0.62		0.83		**0.005**		0.36		0.38	
Fibrosis	0.95		0.92		0.66		0.5		0.99		0.5	

cRORA, complete retinal pigment epithelium pigment and outer retinal atrophy; iRORA, incomplete retinal pigment epithelium pigment and outer retinal atrophy. Significant values are showed in bold.

The nAMD groups N and R were different in terms of treatment duration and number of anti-VEGF injections received. However, there was no association seen between treatment duration or number of injections and any of the five molecular biomarkers.

Additional factors that were unbalanced between the groups, did not show any association with the molecular biomarkers either: presence or absence of cRORA and iRORA, intraretinal or subretinal fluid in the recurrences, and CRT.

Out of the factors that were balanced between the groups, there was a constant negative correlation seen with the subretinal choroidal thickness, significant for sVCAM-1, IL-12p40, and HGF. Occasional correlations were seen for age (with IL-12p40 and HGF, positive correlation), VA improvement (with IL-6, negative correlation), and the CNV type (with PAI-1, higher values for type 2 neovascularisation). However, no association was seen with fibrosis, reticular pseudodrusen. In addition, VEGF-A did not show any association with any of the investigated parameters.

Correcting each clinical parameter for false discovery rate (6 molecules tested), the results remaining significant included the subfoveal choroidal thickness (for sVCAM-1, IL-12p40, and HGF), and type 2 neovascularization (for PAI-1).

## Discussion

The present study was able to identify aqueous humor molecular biomarkers for nAMD patients with incomplete anti-VEGF responses in comparison to nAMD patients with optimal responses and our control group. The double approach, which used proteomics and a specific molecular analysis, showed the involvement of the inflammatory response, complement cascade activation, cytolysis, protein-lipid complex, and vasculature development pathways. In comparison with normally responding nAMD, the incomplete responders group showed particularly increased signals for sVCAM-1, IL-12p40, IL-6, PAI-1, and HGF. Additionally, we were able to identify several biomarkers involved in both nAMD groups, including VEGF-A, MCP-1, and IL-7. A correlation analysis with clinical biomarkers suggested an association with thinner subfoveal choroidal thickness (higher values of sVCAM-1, IL-12p40, and HGF).

Testing multiple proteins, as in our multiplex analysis, is associated with a risk of rejecting the null hypothesis due to a chance result. However, applying a statistical control strategy reduces the sensitivity of the analysis. In the context of precious biological material, ethical considerations apply as well. For this reason, exploration analyses on biological material do not necessarily require controlling for multiple testings. In our study, we applied the false discovery control in order to show the solidity of the results. With this strategy, four out of the five significant results in multiplex analysis (*p* < 0.05) remained significant (sVCAM-1, IL-6, PAI-1, and HGF). The fifth analyte (IL-12p40) was thus not significant. However, as the discussion will show below, biological pathways naturally link this molecule to the other results, and correlation coefficients between the molecules were high. Thus, the biological plausibility of a true rejection of the null hypothesis for IL-12p40 increases the likelihood that it remains a biologically significant result.

VEGF-A is considered to be at the center of all neovascular processes, and anti-VEGF treatment is the confirmed treatment approach. Therefore, the corresponding analyses of VEGF-A were used in our study to confirm the overall approach. The proteomic results confirmed the implication of VEGF-A in both nAMD groups, without significant difference between them. Indeed, total VEGF-A is detected by a proteomic approach which is performed in denaturing condition (Figure 1A). Results obtained by dosages of free VEGF-A performed in non-denaturing condition (multiplex or AlphaLISA analyses) are challenging to interpret because VEGF-A inhibitors interfere within the assays. The interference is depending on the test chosen (antibodies used for the ELISA) and the anti-VEGF agent used for the treatment (interacting differently with VEGF-A) (Waskull et al., 2018). The comparison of free VEGF-A level in the patient treated either with Aflibercept or Ranibizumab is difficult, if not impossible, because of different interactions of the drug with VEGF-A. In keeping with this hypothesis, we found completely different levels of VEGF-A when applying VEGFA AlphaLISA, using different antibodies than the multiplex assay. Actually, VEGF-A became undetectable with AlphaLISA assay (Figure 4B). In addition, Supplementary Figures S2B, C, clearly show that anti-VEGF-A (aflibercept or ranibizumab) disrupt the signal, in both multiplex or AlphaLISA assays, when VEGF-A is measured in control AH patients. Therefore, an antibody-independent approach such as proteomics is highly valuable for total VEGF-A quantitation (Figure 1A).

Interestingly, proteomics (Figure 1B) and multiplex analyses (Table 3; Supplementary Figure S2) detected a VEGFR signal attributable to aflibercept in AH up to 2 months after the last injection; this result may support the hypothesis of the long-term presence of aflibercept in the eye. However, its presence does not necessarily indicate effective anti-VEGF activity as it may be saturated.

Other pathways were of particular interest to this study because they could potentially be responsible for residual fluid despite anti-VEGF treatment. The inflammatory pathway is one of the most promising candidates for explaining residual exudation. Thus, our proteomics results revealing a strong involvement of inflammatory, immunologic, and complement activation pathways in group R are intriguing. In addition, the multiplex analysis results identified several specific molecules implicated in an inflammation process in those patients. In previous studies, several cytokines and angiogenic factors were measured in nAMD patients (without focusing on the response subgroups); among them, sVCAM-1, IL-6, and IL-12p40 were increased in AH of nAMD patients (Jonas et al., 2010; Jonas et al., 2012). Interestingly, we detected a significant increase in sVCAM-1, IL-6, and IL-12p40, particularly in the group with incomplete anti-VEGF response. As part of an inflammatory process, they could provoke exudative activity not responding to anti-VEGF treatment. Interestingly, the pro-inflammatory cytokine IL-6 has been associated with the progression of nAMD (Seddon et al., 2005). It has also been shown to be elevated in the plasma of patients with dry and wet AMD (Nielsen et al., 2019) and during the late stage of AMD, leading to geographic atrophy (Zofia et al., 2019). The observed role of IL-6 in incomplete responders is in accordance with the study results of Chalam and colleagues who showed the correlation of aqueous IL-6 with central subfield macular thickness and an increase in IL-6 cytokine in patients who respond less to bevacizumab treatment (Chalam et al., 2014). However, another study did not confirm a difference in good and poor responders to aqueous IL-6 (Pongsachareonnont et al., 2018), possibly related to the small number of included cases.

In addition to the IL-6 increase, our study revealed a significant increase in sVCAM-1, particularly in incomplete responders. Interestingly, this molecule is known to be directly regulated by IL-6 (Wei et al., 2018) and to have a role in the immune response via IL-12p40 production (Stanley et al., 2008). Thus, it is not surprising that we found increased IL-12p40 in group R as well, although not clearly confirmed after controlling for multiple testing. However, it has been previously associated with early AMD (Klein et al., 2014) and with nAMD patients (Jonas et al., 2012). We hypothesize that an anti-inflammatory treatment targeting either IL-6 or sVCAM-1 could be a promising adjuvant treatment for anti-VEGF incomplete responders. Interestingly, a recent publication has reported adjuvant dexamethasone use to be structurally effective in cases with incomplete anti-VEGF response (Calvo et al., 2014). However, given the potential steroidal side effects, there is a need for well-designed prospective studies to help identify the most promising candidates for this approach.

In addition to inflammation, we observed a significant increase in vasoproliferative factors, such HGF and PAI-1, in the refractory group R as compared to the optimal response nAMD group N. HGF is involved in blood vessel formation via its receptor c-Met; this process is VEGF-independent (Cai et al., 2000). Moreover, a recent study showed an increase in many angiogenic factors, including HGF, after an intravitreal injection of bevacizumab (Cabral et al., 2017). Similarly, our proteomic analysis found an increase in HGF activator (HGFAC), a molecule with the capacity to upregulate HGF (Figure 2B). In addition to HGF, we also noted an increase in PAI-1, which has been reported to have proangiogenic activity in choroidal experimental neovascularization (Lambert et al., 2001). Interestingly, HGF can upregulate PAI-1, at least in human liver-derived HepG2 cells (Imagawa et al., 2006). Even if aqueous PAI-1 seems to be generally unchanged in AMD patients (Bertelmann et al., 2013), it could be upregulated in response to anti-VEGF treatment. Our finding that both PAI-1 and HGF were mainly associated with incomplete anti-VEGF responses suggests that these VEGF-independent pathways are co-responsible for residual fluid, whether this is part of a particular phenotype of nAMD, or a secondary upregulation in response to anti-VEGF treatment. Therefore, both HGF and PAI-1 could be new potential targets of treatment for patients with nAMD responding poorly to anti-VEGF.

In our results, treatment with aflibercept vs. ranibizumab seemed to influence the molecular concentrations described before. However, the study was not designed for this subgroup analysis, and the numbers were too small for conclusive results. In the light of our previous discussion regarding drug interactions and test results, we considered using AlphaLISA for replication of the multiplex results for sVCAM (Supplementary Figure S4). In fact, we found similar increases in both aflibercept-treated and ranibizumab-treated patients within group R, contrasting with the difference seen on multiplex tests. Therefore, the role of the anti-VEGF agent was not clear. The available material did not allow for further investigations of this issue.

However, the issue about incomplete anti-VEGF response, often called refractory nAMD, is complex. There are a number of open questions to investigate, which might finally become relevant to the interpretation of our results. First, incomplete responders are likely to be a inhomogeneous group. Besides inflammatory and vasoproliferative pathways, there are potential factors such as drug tolerance, polypoidal choroidal vasculopathy, large pigment epithelium detachments, and degenerative changes which might play a role (Yang et al., 2016). Furthermore, the residual fluid observed at monthly visits might show some dynamic changes within the first month, as the maximum response often occurs earlier than at 1 month (Bontzos et al., 2019). Thus, besides inhomogeneity within incomplete responders, there might be a continuous spectrum of the degree of response. In order to eliminate borderline cases, the study required at least 6 months of persistent fluid for group R. Furthermore, clinical biomarkers were investigated for their potential association with higher molecular biomarkers. The groups R and N differed in terms of mean duration of preceding treatment and number of anti-VEGF injections received, the mean visual acuity change, as well as for the distribution of intraretinal vs. subretinal fluid, central retinal thickness, and atrophic changes. However, none of these showed any association with the molecular biomarkers. On the other hand, out of the balanced clinical factors, there was a strong suggestion for thinner choroidal thickness being associated with higher concentrations of sVCAM-1, IL-12p40, and HGF, thus implying both inflammatory and vasoproliferative molecules. This contrasts with the absence of association with reticular pseudodrusen, which are known to be associated with thinner choroid. However, future studies should focus on the role of the choroid, and particularly the choriocapillaris. Thinning might go along with ischemia of the RPE, and induce more inflammatory and vasoproliferative response.

Limitations of the present study include the relatively small number of patients in each group and the failure to obtain reliable measurements for a subset of targeted molecules. Furthermore, the study was not designed to identify the influence of the anti-VEGF agent. Therefore, any conclusions regarding these subgroups need to be handled with care. The control group was not completely age-matched with the both nAMD groups and also showed a mild imbalance of sex distribution. However, the molecules with significant differences between the groups are not known to be age- or sex-dependent. In addition, the focus of this study was the difference between groups N and R, and these were both age- and sex-matched.

In conclusion, using the proteomics and multiplex analysis, we found that patients with nAMD incompletely responding to anti-VEGF treatment showed a significant increase in molecular markers in AH associated with inflammatory response and neovascularization/vascular development pathways. In addition, proteomic analyses also described the implication of complement cascade activation, cytolysis, and the protein-lipid complex. The multiplex approach highlighted specific molecules, undetectable by the proteomics approach, that were involved in the angiogenic process (HGF, PAI-1) and the inflammatory response (sVCAM-1, IL-6, IL-12p40). The causal relationship with residual fluid will have to be confirmed by further studies, with the final aim to define new therapeutic targets to improve the outcomes of nAMD with incomplete anti-VEGF response. The latter could include steroids as well as more specific targeting of molecules such as found in our study to be implicated in incomplete anti-VEGF response.

## Data Availability Statement

The mass spectrometry proteomics data have been deposited to the ProteomeXchange Consortium via the PRIDE partner repository with the dataset identifier PXD022471.

## Ethics Statement

The studies involving human participants were reviewed and approved by the local ethics committee (CER-VD, protocol ID 2017-02175). It adhered to all national legal requirements and the tenets of the Declaration of Helsinki.. The patients/participants provided their written informed consent to participate in this study.

## Author Contributions

MI, GJ, FE, KO, and DY: human sample collection. MI and GJ: clinical characterization of patients. MI, RR, and SC: study protocol. WP: proteomic analysis. BA: multiplex analysis. SC: sample management, biobank. BF and MF: statistical analyses. MI and RR: conception and design of the study; interpretation of data; manuscript writing; final revision approval of the manuscript.

## Funding

RR is supported by the Gelbert Foundation and the “Art & Vie” Foundation. The study was supported by an internal grant of the Foundation “Asile des aveugles.”

## Conflict of Interest

The authors declare that the research was conducted in the absence of any commercial or financial relationships that could be construed as a potential conflict of interest.
