# Supplementary material for: Molecular Biomarkers of Neovascular Age-Related Macular Degeneration With Incomplete Response to Anti-Vascular Endothelial Growth Factor Treatment
Source: Front Pharmacol. 2020 Dec 29;11:594087. doi: 10.3389/fphar.2020.594087 (PMC7802772; doi:10.3389/fphar.2020.594087)
Supplement: Supplementary file 2 [file datasheet2.docx]

***Supplementary material***

**Molecular biomarkers of neovascular age-related macular degeneration with incomplete response to anti-vascular endothelial growth factor treatment.**

Mantel Irmela^1^*, Borgo Angelica^1^, Guidotti Jacopo^1^, Forestier Edwige^1^, Kirsch Olga^1^, Derradji Yasmine^1^, Waridel Patrice^3^, Burdet Frédéric^2^, Mehl Florence^2^, Schweizer Claude^1^ and Roduit Raphaël^1^*

^1^ Department of Ophthalmology, University of Lausanne, Jules-Gonin Eye Hospital, Lausanne, Switzerland. ^2^ Swiss Institute of Bioinformatics, Lausanne, Switzerland. ^3^ Protein Analysis Facility, University of Lausanne, Lausanne, Switzerland.

***** **Correspondence:**Dr Irmela Mantel, Jules-Gonin Eye Hospital, Av. de France 15, CH-1002 Lausanne, Tél: +41 21 626 85 89, Fax: +41 21 626 54 55, [irmela.mantel@fa2.ch](mailto:irmela.mantel@fa2.ch)

Dr Raphaël Roduit, Jules-Gonin Eye Hospital, Av. de France 15, CH-1002 Lausanne, Tél: +41 21 626 82 13, Fax: +41 21 626 54 55, [raphael.roduit@fa2.ch](mailto:raphael.roduit@fa2.ch)


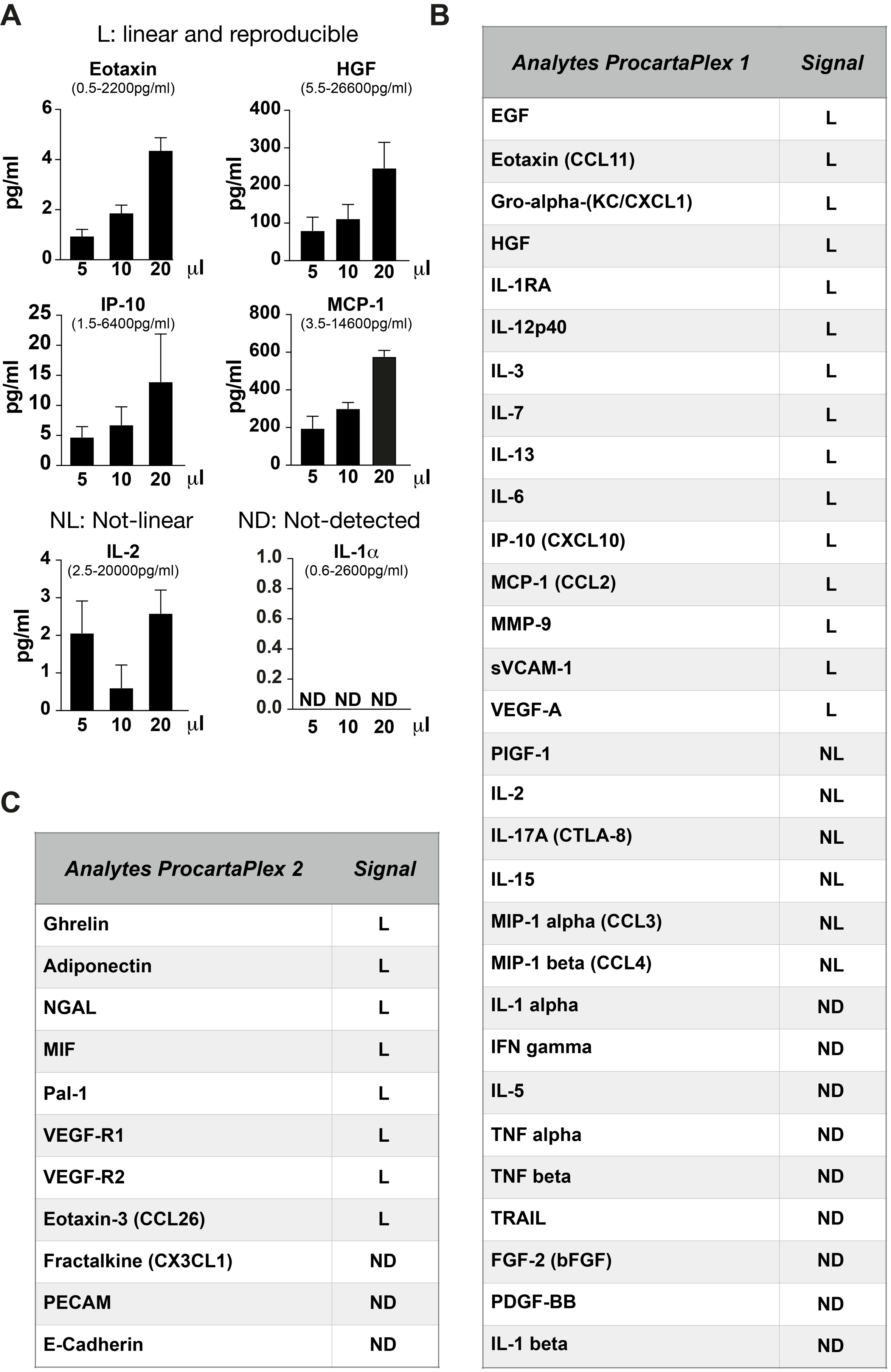


**Supplemental Fig.1: Setup of Multiplex assay. A)** Examples of linearity tests with 5, 10 and 20 ul of AH from 2 controls. EOTAXIN, HGF, IP-10 and MCP-1 had a linearity response and each dilution was in the standard curve (written below each metabolite); IL-2 showed a non-linear response close to the detection limit; IL-1α was below the detection limit. **B)** List of metabolites used in the first round of analysis with ProcartaPlex 1 to analyze the linearity response (5, 10 and 20ul of AH) and **C)** New metabolites added in the ProcartaPlex 2 and used in the second round analysis. (L) linear, (NL) non-linear and not detected (ND)

**Supplemental Fig.2: Heatmap representation of proteomic data in AH from nAMD patients with normal response (N), incomplete response (R), and controls (C).** We analyzed 10 samples per group using 15ul of AH.


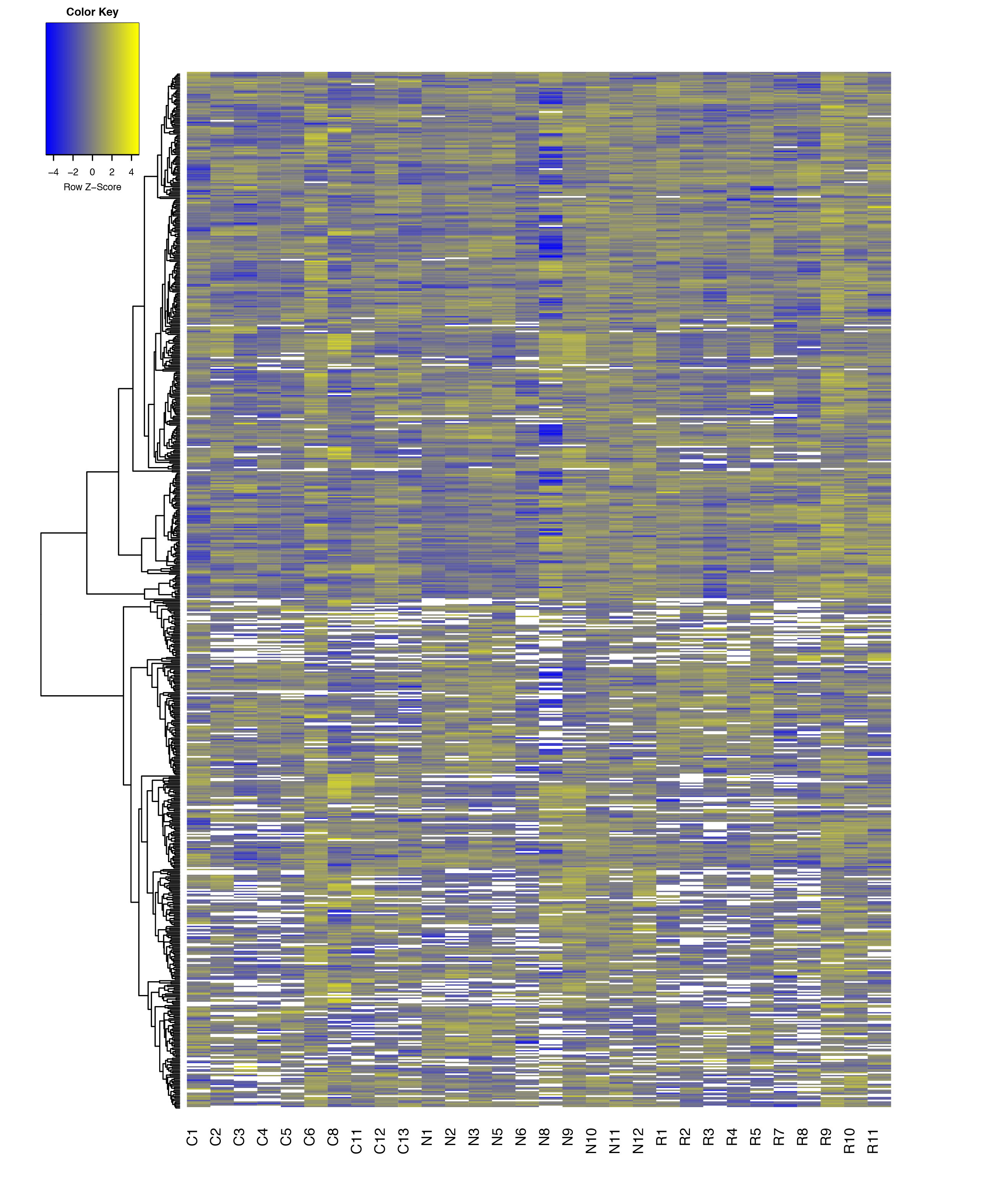


.


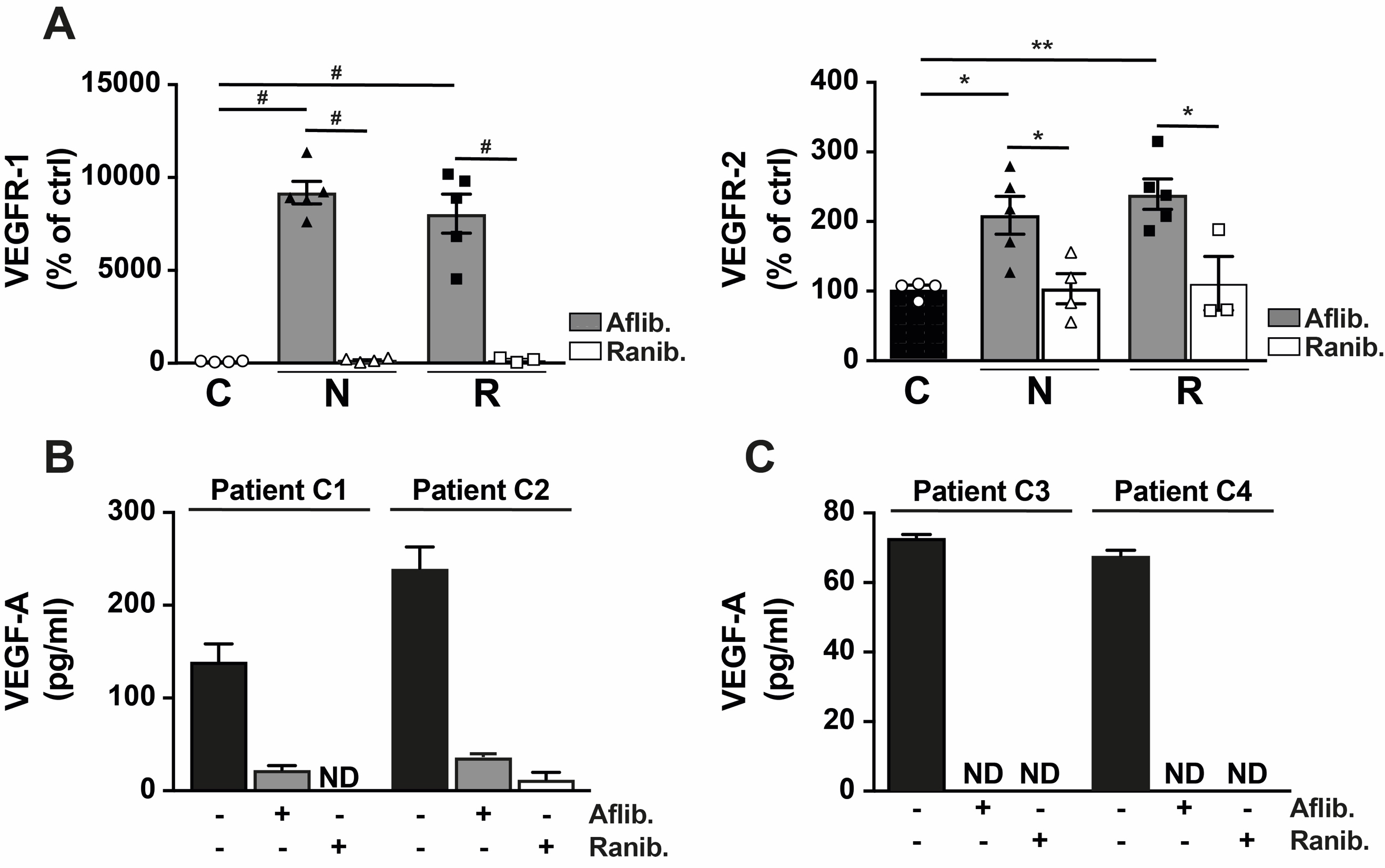


**Supplemental Fig.3: Dosage of VEGFR-1, VEGFR-2 and VEGFA.** **A)** Multiplex analysis of VEGFR-1 and VEGFR-2 in AH of the three different groups of patients depending of the treatment (aflibercept: Aflib. or ranibizumab: Ranib.). Results are expressed as mean ± SEM and as % of the control (* p < 0.05, ** p<0.009 and # p<0.0005, using ANOVA Holm-Sidak's multiple comparisons test per analyte). **B)** Dosage of VEGF-A by multiplex analysis using AH of control patients, with or without aflibercept (Aflib.) or ranibizumab (Ranib.). **C)** Dosage of VEGF-A by AlphaLISA analysis using AH of control patients, with or without aflibercept (Aflib.) or ranibizumab (Ranib.).

**Supplemental Fig.4: Schematic representation of multiplex results showed in table 2 according to the drug.** Dosage of sVCAM-1, HGF, PAI-1 and IL12p40 **(A)**, EOTAXIN and MMP-9 **(B)**. MCP-1 and IL-7 **(C)** by multiplex analysis using AH patients from the three different groups. Results are expressed as mean ± SEM and as % of the control (* p < 0.05, ** p<0.005 and *** p<0.0005, using ANOVA Holm-Sidak's multiple comparisons test per analyte).


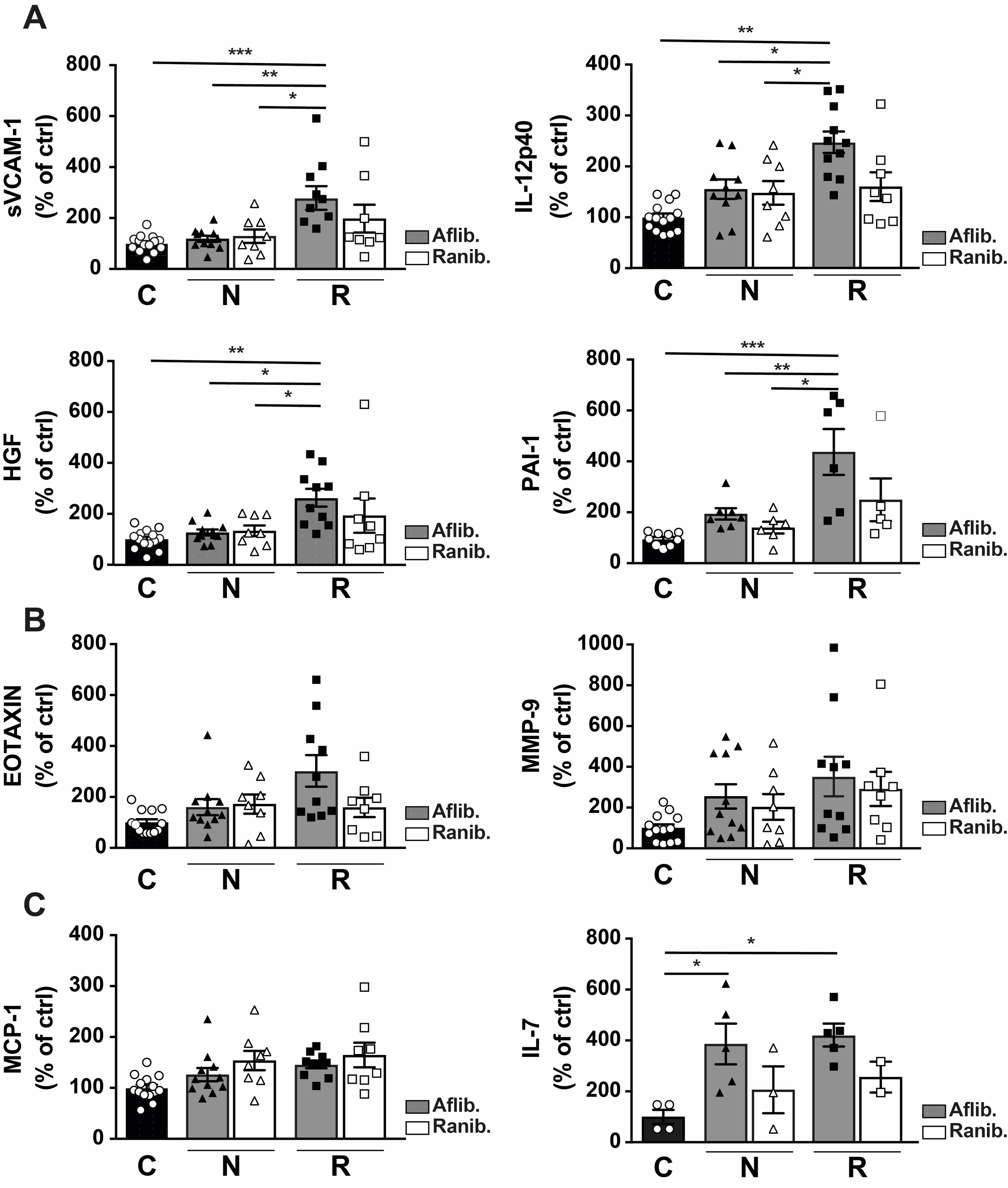


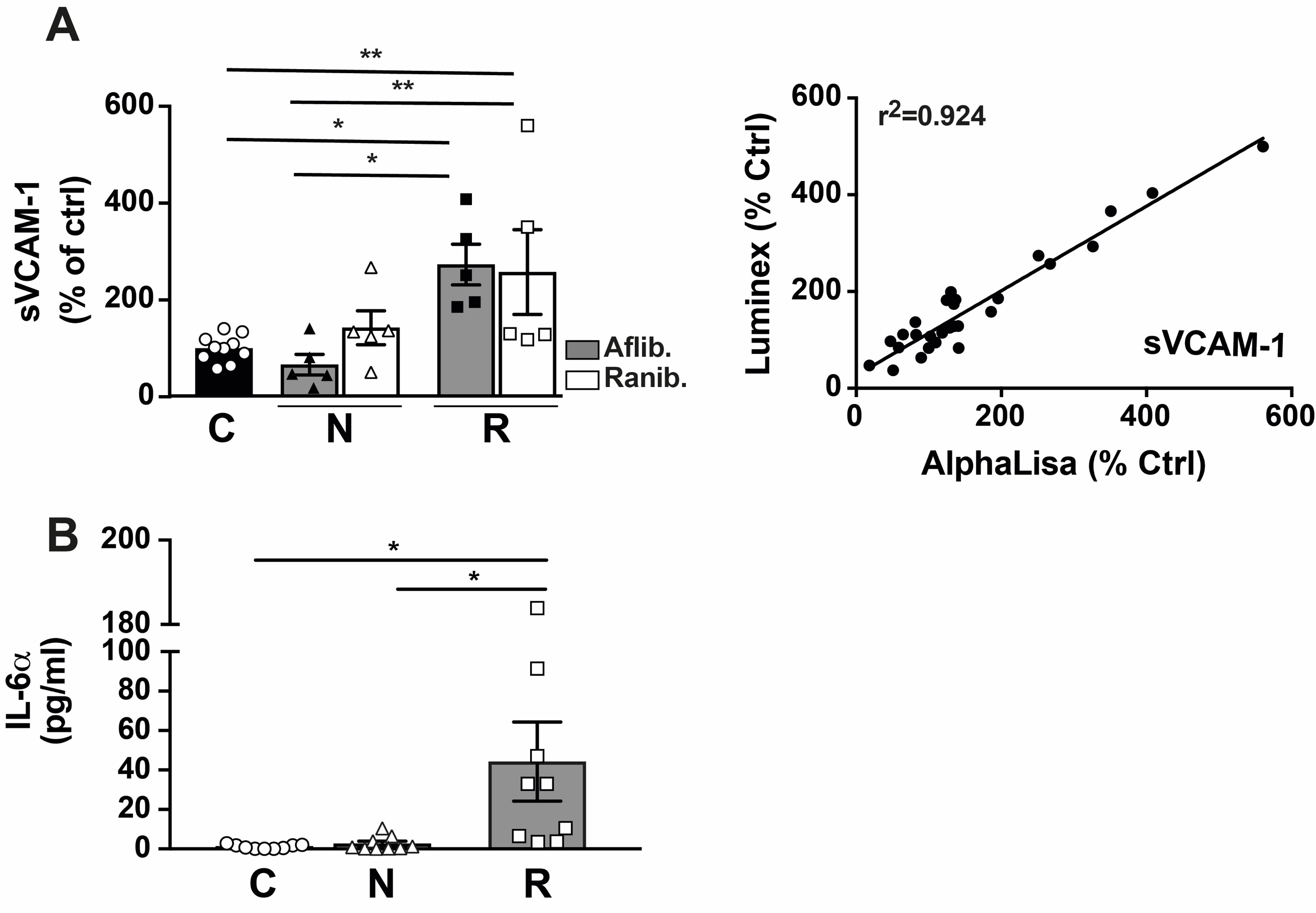


**Supplemental Fig.5: AlphaLISA analysis. A)** Dosage of sVCAM-1 by AlphaLISA in AH of the three different groups of patients depending of the treatment (aflibercept: Aflib. or ranibizumab: Ranib.). Results are expressed as mean ± SEM and as % of the control (* p < 0.05, ** p<0.005, using ANOVA Holm-Sidak's multiple comparisons test per analyte). Comparison of sVCAM-1 dosage by multiplex and AlphaLISA analysis. **B)** Dosage of IL-6 by AlphaLISA in AH of the three different groups of patients. Results are expressed as mean ± SEM and as % of the control (* p < 0.05, using ANOVA Holm-Sidak's multiple comparisons test per analyte)

**Supplemental proteomic data** (Excel file: SupplementalData1.xlsx)
